# Clinical significance of asymmetric venous vasculature on minimum-intensity projection in patients with moyamoya disease

**DOI:** 10.1097/MD.0000000000031067

**Published:** 2022-10-14

**Authors:** Min Jeong Han, Sun Jun Kim

**Affiliations:** a Department of Pediatrics, Jeonbuk National University Medical School, Jeonbuk, Korea; b Research Institute of Clinical Medicine of Jeonbuk National University, Jeonbuk National University Medical School, Jeonbuk, Korea; c Biomedical Research Institute of Jeonbuk National University Hospital, Jeonbuk National University Medical School, Jeonbuk, Korea

**Keywords:** cerebral infarction, children, minimum intensity projection, moyamoya disease

## Abstract

This study analyzed the clinical significance and characteristics of asymmetric venous blood flow in patients with Moyamoya disease (MMD) using minimum intensity projection (minIP) susceptibility-weighted imaging. The minIP views of 30 patients diagnosed with MMD were retrospectively analyzed using clinical features, brain magnetic resonance angiography, electroencephalography, and brain single-photon emission computed tomography (SPECT). Simultaneously, differences between patients with acute cerebral infarction and non-MMD causes were analyzed. Twelve (40.0%) of the 30 patients had asymmetrical venous flow, which is usually seen in patients with acute cerebral infarction (*P* = .146). They also had significantly higher Suzuki stages than symmetric patients (*P* = .014), with five (41.7%) and three (25.0%) of them in stages 4 and 5, respectively. When the Suzuki stages of both hemispheres were different, more veins were found in the stenotic hemisphere (88.9%). Brain SPECT showed more severe hypoperfusion on the side with prominent vascularity in the minIP view (100.0%). Additionally, asymmetric blood flow was observed in 66.7% of the patients with cerebral infarction caused by MMD, whereas only 11.1% of the children with cerebral infarction caused by non-MMD had asymmetry (*P* = .005). Patients with MMD showed asymmetric hypointensity of the cortical veins with a minIP appearance. The venous structure showed greater signal loss on SWI and was more prominent in the hemisphere where stenosis was advanced or infarction occurred in other examinations. Cerebral infarction in patients with MMD tended to occur with asymmetrically prominent venous patterns with damaged areas in minIP images, which had distinct characteristics from those of patients without MMD.

## 1. Introduction

Moyamoya disease (MMD) is a chronic progressive cerebrovascular occlusive disease.^[1]^ It was first reported in 1957 by Takeuchi as hypoplasia of bilateral internal carotid arteries.^[2]^ MMD mainly affects the distal internal carotid artery, proximal branches of the anterior and/or middle cerebral arteries, and a few posterior cerebral arteries.^[3,4]^ In children, the clinical symptoms of MMD typically include acute cerebrovascular events such as transient ischemic attacks, cerebral artery infarctions, and seizures.^[5,6]^ Hence, the existing approach to MMD focuses on the evaluation of the arterial vessels. The most commonly used diagnostic methods include magnetic resonance imaging (MRI) and MR angiography (MRA).^[7]^ Brain perfusion single-photon emission computed tomography (SPECT) is also used to demonstrate preoperative impaired perfusion or postoperative effects of revascularization surgery.^[8–11]^ MMD is associated with cortical microvascularisation and basal collaterals to compensate for arterial stenosis, which is a specific finding.^[12]^ These compensatory changes in arterial blood flow are expected to cause a corresponding change in the venous structure. However, few studies have been conducted on the venous patterns in the brains of patients with MMD.

Susceptibility-weighted imaging (SWI) of brain MRI is the best imaging technique for detecting iron deposition. It has a blood-oxygen level-dependent effect, such that the signal strength of such images depends on the oxygen saturation of the blood.^[13,14]^ SWI generates an image that is sensitive to deoxygenated blood. It can be mapped over multiple image planes (typically 5–10 image planes) using minimum-intensity projections (minIP). These minIP images can reveal the venous structure by attenuating the signal received from the brain tissue,^[15]^ thus allowing the continuity of lesions or venous vessels through the image plane and acquisition of a more detailed image of distorted vein blood vessels.^[16]^ In 2017, a study was conducted on the pattern of cerebral veins according to oxygen saturation before and after transposition of the great arteries (TGA).^[17]^ In this study, changes in cerebral veins were observed according to oxygen saturation before and after anterior aortic surgery. Neonates with TGA showed a higher prominence of the cerebral vein in the minIP of SWI when they had worse deoxyhaemoglobin levels and/or lower arterial blood oxygen saturation. In 2011, through an animal experiment, Masanori et al observed venous flow patterns when M1 occlusion was performed during surgery.^[18]^ Animals showed diminished venous flow, such as darkening of the venous blood, small vessel collapse, and reduced but incompletely occluded venous flow. However, when the M1 supply is not immediately shut off and progresses gradually, such as in intracranial atherosclerosis, collateral compensation for stabilizing cerebral blood flow is achieved through arterial collateral development, as well as venous autoregulation.^[19,20]^ Based on these findings, we hypothesized that the oxygen extraction fraction for compensation would be increased in the direction of more advanced arterial stenosis in MMD patients, following the changes in deoxyhemoglobin concentration that would appear through minIP. We also assumed that venous changes would be more prominent in patients with MMD and acute infarction.

This study aimed to analyze the clinical significance and characteristics of asymmetric vascularity in minIP images of patients with MMD. In addition, we compared the characteristics of acute cerebral infarctions caused by MMD and non-MMD infarctions.

## 2. Subject and Methods

### 2.1. Participants

This retrospective study was approved by the local institutional review board (CUH 2021-05-038). Data were collected from 2013 to 2020 after the introduction of the minIP technique at Jeonbuk National University Hospital in 2013. During this period, patients who met the current diagnostic criteria for MMD were included^[21]^: stenosis or occlusion of the terminal portion of the intracranial ICA or proximal portions of the anterior cerebral artery and/or the middle cerebral artery; development of abnormal vascular networks near the occlusive or stenotic lesions in the arterial phase; and bilateral lesions. Data from patients without SW images were excluded. We evaluated patient data, such as age of onset for symptomatic cases or detected age for asymptomatic cases; sex; clinical symptoms such as headache, hemiparesis, or seizure; and MRI, MRA, electroencephalography (EEG), and brain SPECT findings.

**Figure 1.**
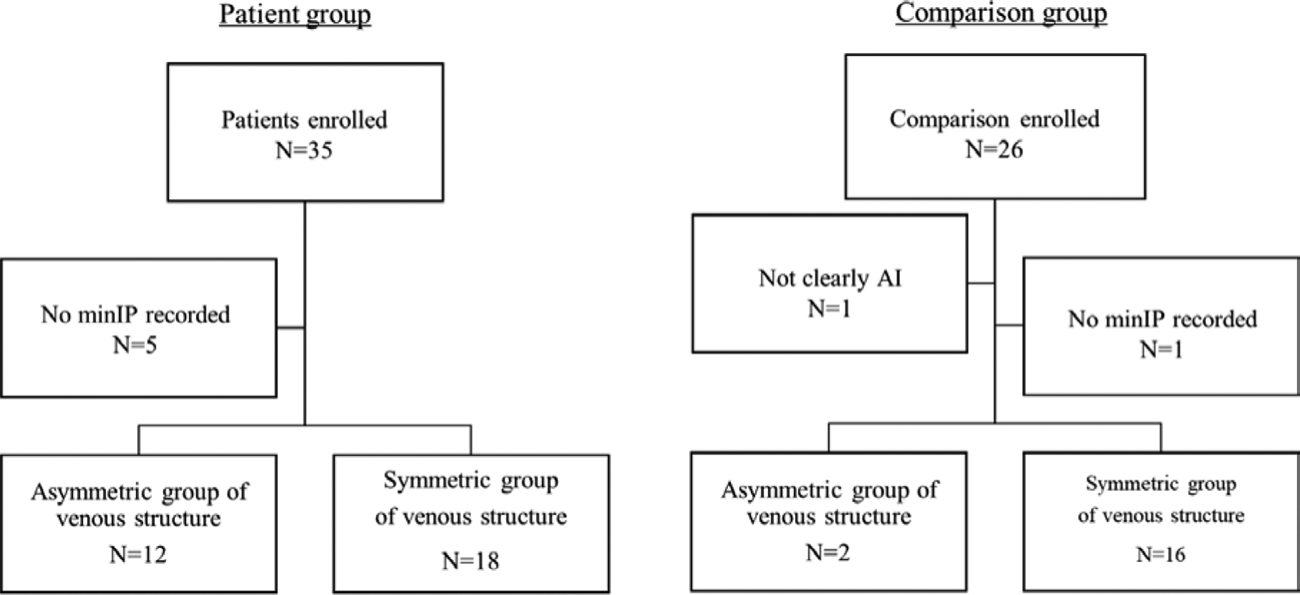
Diagram of the inclusion protocol of patients with moyamoya disease and other acute cerebral infarctions included in this study. AI = Acute infarction, minIP = minimum intensity projection, MMD = moyamoya disease.

A total of 35 patients with MMD were enrolled, and minIP images of the brains of 30 patients were analyzed (Fig. 1). If there was an intensity difference between hemispheres, the patient was classified into the asymmetric group. When it was difficult to clearly identify the difference, patients were classified into the symmetric group. Eighteen patients in the symmetric group had grossly symmetric bilateral venous flow distribution in the minIP, whereas 12 patients in the asymmetric group showed distinct asymmetric hypointensities in the cortical veins. Patients with acute cerebral infarction and non-MMD causes during the same period were included in the comparison group. Of the 20 patients diagnosed with non-MMD acute cerebral infarction, 2 were excluded because the minIP was not taken and cerebral infarction could not be identified on MRI (Fig. 1). Eighteen patients were included in the control group.

### 2.2. Data sources

The severity of MMD was subdivided into Suzuki stages 1 to 6 according to the degree of stenosis and the development of moyamoya vessels on MRA, which was first introduced by Suzuki in 1969.^[22,23]^ The demographic characteristics, clinical symptoms, presence of acute and/or chronic infarction, and Suzuki stage of patients with MMD were compared between the asymmetric and symmetric groups. In the asymmetric group, asymmetric venous structures in minIP images were analyzed for their association with focal abnormalities on EEG, brain SPECT, or brain MRA as well as clinical symptoms or acute infarction areas. In addition, the probability of patients with unilateral neurological symptoms showing abnormalities in the related hemisphere was compared for each test (SWI, EEG, MRA, and brain SPECT). We also investigated the differences in clinical characteristics and radiologic findings between the MMD and comparison groups.

### 2.3. Statistical methods

Statistical Package for the Social Sciences (version 23.0, Chicago, IL) was used for statistical analyses. The clinical characteristics of the two groups were compared using either the independent samples *t* test for numerical variables or Pearson’s chi-square test for qualitative variables. Statistical significance was set at *P <*.05.

## 3. Results

### 3.1. Clinical characteristics of patients

Between January 2013 and December 2020, 30 patients (20 male and 10 female; mean age 98.0 ± 49.7 months at the time of MRI acquisition) were enrolled in this study. Among the 30 patients, 12 were in the asymmetric group and 18 were in the symmetric group. Both groups had more male patients than female patients (72.2% and 58.3%, respectively), with the average age being 99.3 ± 50.1 months in the symmetric group and 96.1 ± 49.0 months in the asymmetric group (Table 1). There were no differences in the clinical symptoms, such as general weakness, hemiparesis, seizures, and dizziness between the two groups, and there were no differences in terms of family history (Table 1).

**Table 1:** Clinical characteristics between the asymmetric and symmetric groups.

	Total (n = 30)	Symmetry (n = 18)	Asymmetry (n = 12)	*P* value
Sex				.755
Female	10 (33.3)	5 (27.8)	5 (41.7)	
Male	20 (66.7)	13 (72.2)	7 (58.3)	
Age (mo)	98.0 ± 49.7	99.3 ± 50.1	96.1 ± 49.0	.609
General weakness (%)	10 (33.3)	8 (44.4)	2 (16.7)	.122
Hemiparesis (%)	16 (53.3)	10 (55.6)	6 (50.0)	.775
Seizure (%)	8 (26.7)	5 (27.8)	3 (25.0)	.872
Dizziness (%)	3 (10.0)	3 (16.7)	0	.465
Headache (%)	13 (43.3)	7 (38.9)	6 (50.0)	.563
Family history (%)	4 (13.3)	3 (16.7)	1 (8.3)	.723
Acute infarction (%)	6 (20.0)	2 (11.1)	4 (33.3)	.146
Chronic infarction (%)	5 (16.7)	5 (27.8)	0	.047*
Suzuki stage (%)				
1	0	0	0	
2	6 (20.0)	4 (22.2)	2 (16.7)	
3	13 (43.3)	11 (61.1)	2 (16.7)	.014*
4	8 (26.7)	3 (16.7)	5 (41.7)	
5	3 (10.0)	0	3 (25.0)	
6	0	0	0	

Values are number of cases (%). Mean values are presented with standard deviations.

### 3.2. Neuroimaging findings

**Figure 2.**
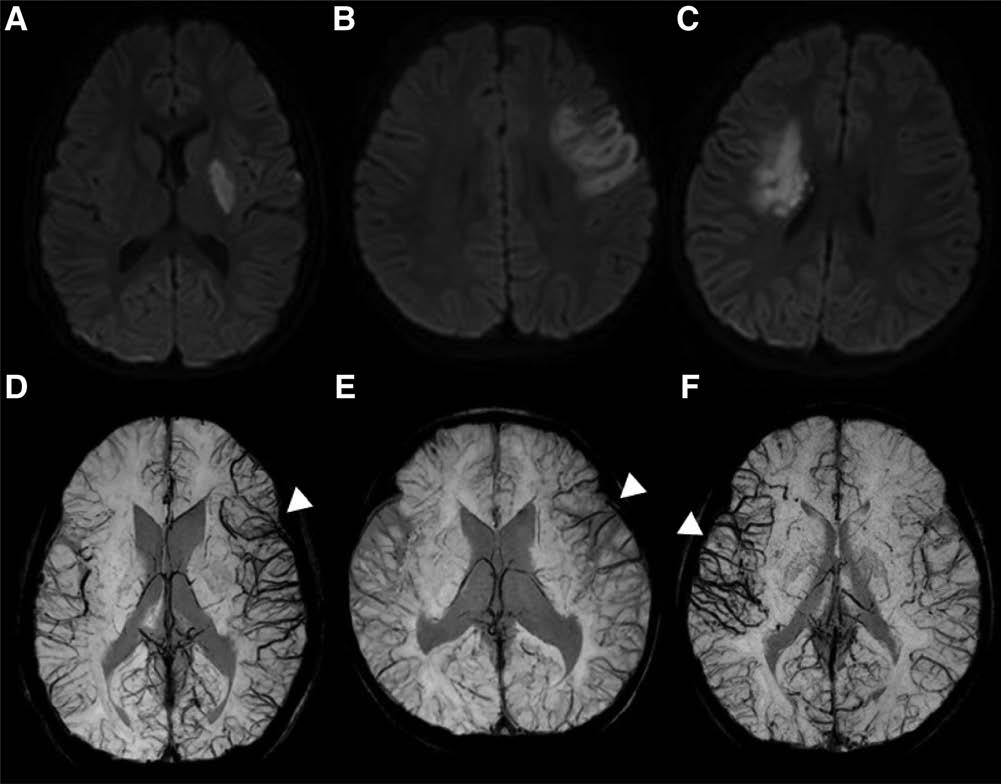
Comparison of asymmetric venous structures in minimum intensity projection and diffusion weighted images for acute infarction in patients with moyamoya disease. A b = 1000 diffusion-weighted images (DWI) of patients with acute infarction in the asymmetric group showing restricted diffusion values in the infarcted area (A, B, and C). A minimum intensity projection (minIP) image of each patient shows increased hypointense vascularity (arrowheads) on the affected side, especially in the damaged area (E, F, and G).

In brain MRA, the Suzuki stage distribution across the two groups was significantly different (*P* = .014, Table 1). In the asymmetric group, the Suzuki stage was more advanced than in the symmetric group: five patients (41.7%) had stage 4, while three patients (25.0%) had stage 5. In the symmetric group, 11 patients (61.1%) had stage 3, and four patients (22.2%) had stage 2 disease. Acute infarction was confirmed in six patients (20%) among those who had MMD, in four patients (33.3%) in the asymmetric group, and in two patients (11.1%) in the symmetric group; however, the difference was not statistically significant (*P* = .146). All four patients with acute infarction in the asymmetric group showed markedly increased venous structures in the hemisphere where the infarction occurred, especially in the damaged areas on diffusion-weighted images (DWI) (Fig. 2).

### 3.3. Clinical and radiologic correlations with asymmetric vascularity in minip images

In the asymmetric group, eight children showed decreased focal brain activity or epileptiform discharges on EEG. All patients (100%) showed greater signal loss in SWI in the hemisphere with EEG abnormalities (Table 2). Only 4 patients underwent brain SPECT, and all of them (100%) showed significantly more vascularity on the side with more hypoperfusion on SPECT. When the Suzuki stages of both hemispheres were different, venous hypervascularity was observed in the stenotic hemisphere (88.9%) (Table 2). Meanwhile, brain SPECT had the highest sensitivity, with images showing lesions matching the symptoms when patients with MMD presented with unilateral neurologic symptoms (Table 3). When SPECT was performed on patients with unilateral symptoms, 85.7% of the patients had confirmed related lesions. The minIP images showed related unilateral hypervascularity on the side of the advanced stenosis in 38.9% of patients, with the same sensitivity as that of EEG and brain MRA.

**Table 2:** The concordance rate between the direction of increased vasculature in minIP and other examinations.

	Asymmetric patients	minIP direction correlation patients
Acute unilateral symptom	8	7 (87.5%)
EEG* asymmetric BGA* or epileptiform discharge	8	8 (100%)
Acute infarction	4	4 (100%)
SPECT* (total: 4)	4	4 (100%)
Brain MRA* asymmetry	9	8 (88.9%)

Values are number of cases (%).

BGA = background activity, EEG = electroencephalography, MRA = magnetic resonance angiography, SPECT = single photon emission computed tomography.

**Table 3:** Comparison of the sensitivity of minimum intensity projection and other examinations in moyamoya disease patients with clinically unilateral neurological symptoms.

N (total 18)	Asymmetry	Direction matched with examination (sensitivity, %)
Brain MRI*, acute infarction	6	6 (33.3%)
EEG*	9 (50.0)	8 (44.4)
Brain MRA*	8 (44.4)	7 (38.9)
Brain minIP* asymmetry	8 (44.4)	7 (38.9)
SPECT* (n = 7)	6 (85.7)	6 (85.7)

Values are number of cases (%).

EEG = electroencephalography, minIP = minimum intensity projection, MMD = moyamoya disease, MRA = magnetic resonance angiography, MRI = magnetic resonance imaging, SPECT = single photon emission computed tomography.

### 3.4. Comparison of patients with acute cerebral infarction from MMD and non-MMD causes

Six children had cerebral infarction due to MMD and 18 patients had infarction due to other causes during the same study period (Table 4). There were no significant differences in the most common clinical symptoms, except for hemiparesis, between the two infarction groups. Patients with cerebral infarction due to MMD showed asymmetry of minIP in 66.7% (n = 4) of cases, while only 11.1% (n = 2) of cerebral infarction patients without MMD had visible asymmetry in minIP images (*P* = .005). However, the EEG findings showed no difference (*P* = .541).

**Table 4:** Comparison between patients with acute infarction caused by moyamoya disease and non-moyamoya disease causes.

	Total	Moyamoya with AI* (total 6)	Other AI (total 18)	*P* value
Sex				.063
Male	12	5 (83.3)	7 (38.9)	
Female	12	1 (16.7)	11 (61.1)	
Age (mo)	54.6 ± 65.1	57.2 ± 28.1	49.3 ± 73.3	.065
General weakness (%)	3	2 (33.3)	1 (5.6)	.343
Hemiparesis (%)	8	4 (66.7)	4 (22.2)	.048
Seizure (%)	11	2 (33.3)	9 (50.0)	.5
Dizziness (%)	1	0	1 (5.6)	.871
Headache (%)	5	2 (33.3)	3 (16.7)	.406
Family history (%)	1 (n < 5)	1 (16.7)	0	.581
minIP*	24			.005
Asymmetry (%)	6	4 (66.7)	2 (11.1)	
EEG* total	21	6	15	.541
Asymmetry (%)	16	4 (66.7)	12 (80.0)	

Values are number of cases (%). Mean values are presented with standard deviations.

AI = acute infarction, EEG = electroencephalography, minIP = minimum intensity projection.

## 4. Discussion

We found that the cerebral vein became more prominent in the direction where stenosis was more advanced in patients with MMD on SWI. Based on these observations, the following results were obtained: First, Suzuki stage was higher in patients with asymmetric venous vascularity on minIP images (Table 1). Second, the hemisphere where stenosis was more advanced or functionally declined could be verified using minIP, MRA, SPECT, and EEG (Table 2). This indicates that when patients with MMD have distinct asymmetric hypervascularity in one hemisphere in minIP images, most of them also show advanced lesions on the side of increased venous structures on other examinations. We also calculated the sensitivity of each examination in detecting advanced hemisphere damage related to clinical unilateral symptoms (Table 3). When MRI alone was performed, lesions associated with unilateral symptoms were observed in only 33.3% of patients. SPECT was the most sensitive test and detected the condition at a rate of 85.7%. The minIP images of SWI showed the same sensitivity as those of MRA and EEG. Third, in 66.7% (n = 4) of the MMD patients with acute infarction, a distinct cerebral venous structure was observed, especially in the infarcted area (Fig. 2). In addition, patients with MMD and acute infarction showed clearly increased vascularity compared to patients with non-MMD causes (Table 4, *P* = .005). In our study, two patients with acute infarction due to non-MMD also had asymmetric cerebral venous flow. One of them came to our hospital for recurrent arterial infarction, which was later diagnosed as multiple sclerosis, and had received betaferon treatment. The other patient tested positive for homogenous fluorescent antinuclear antibodies, which made autoimmune-induced cerebral infarction a consideration. It is possible that these two patients also had chronic progressive stenosis in the cerebral arteries, suggesting an asymmetric appearance of the cerebral vein, occurring through a mechanism similar to that of MMD patients. Four children with MMD complained of hemiparesis, which was significantly higher than that in other cerebral infarction patients (*P =*.048). We suggest two hypotheses for the occurrence of prominent hypointense vessels in the hemisphere with advanced stenosis. An increase in oxygen consumption due to progressive arterial stenosis may cause increased venous deoxyhaemoglobin levels, making the veins appear larger and more distinct. In particular, when ischemia occurs, pooling of deoxygenated blood can be affected by adjacent veins.^[24]^ Alternatively, as the cortex is chronically exposed to hypoxia, the venous structure may increase as a compensatory mechanism, along with the generation of moyamoya arterial vessels.

MMD treatment is largely divided into medical treatment and surgical revascularization. Antiplatelet agents or calcium channel blockers can be used for treatment; however, 37% of patients manifest symptoms related to nervous system damage, and 3% of patients show deterioration if treated solely with medications, without surgery.^[25,26]^ Thus, bypass surgery for revascularization has generally been accepted as the most effective treatment for MMD.^[27,28]^ Surgical approaches are generally accepted for ischemic MMD compared with hemorrhagic MMD.^[29]^ Therefore, surgical revascularization is important in children with ischemic MMD, consistent with our findings. Many studies have considered the prognosis after surgery, although factors related to outcomes have not been clearly established. A common opinion is that the younger the age, the better is the prognosis after surgery.^[30–33]^ According to a study published in 2019, the prognosis is significantly better for Suzuki stage 4 than for other stages.^[31]^ These results are consistent with those of our study, which showed that the most prominent asymmetric venous flow was found in Suzuki stage 4. Compensatory venous flow could be changed according to the degree of arterial stenosis, and based on the findings of the above-mentioned studies, we can estimate that collateral circulation is most prominent in Suzuki stage 4. Future studies should also discuss the postoperative prognosis according to cerebral venous flow in minIP images.

The strength of this study is that the minIP images of SWI can be used to predict the possibility of advanced stenosis in the hemisphere showing increased venous flow without the need for additional examinations such as MRA, EEG, and SPECT. However, because not all patients show asymmetry, one limitation is that further examinations are required.

There are also additional limitations: the number of patients with MMD was small, and the data were collected retrospectively. Information on patient prognosis was insufficient because those who received surgical treatment were not followed-up. Thus, future studies regarding the role of the minIP view as a predictor of prognosis after treatment, and extensive prospective studies are needed.

## 5. Conclusion

Patients with MMD generate new arterial blood vessels to compensate for progressive chronic cerebral arterial stenosis. In addition, we revealed that venous structures increased for compensation, especially in the hemisphere with more advanced stenosis. When acute cerebral infarction occurs in a patient with MMD, the damage is located on the side with an increased number of venous structures.

## Author contributions

**Conceptualization:** Min Jeong Han, Sun Jun Kim.

**Data curation:** Min Jeong Han.

**Investigation:** Min Jeong Han.

**Methodology:** Sun Jun Kim.

**Supervision:** Sun Jun Kim.

**Validation:** Sun Jun Kim.

**Visualization:** Min Jeong Han.

**Writing – original draft:** Min Jeong Han.

**Writing – review & editing:** Sun Jun Kim.
